# Small villages and their sanitary infrastructure—an unnoticed influence on water quantity and a threat to water quality in headwater catchments

**DOI:** 10.1007/s10661-023-12051-6

**Published:** 2023-11-16

**Authors:** Caroline Spill, Lukas Ditzel, Matthias Gassmann

**Affiliations:** Department of Hydrology and Substance Balance, University of Kassel, Kassel, Germany

**Keywords:** Wastewater treatment plant (WWTP), Nitrate, Ammonium, Phosphorus, High-frequency data, Combined sewer overflow (CSO)

## Abstract

In rural catchments, villages often feature their own, separate urban water infrastructure, including combined sewer overflows (CSOs) or wastewater treatment plants (WWTPs). These point sources affect the water quantity and quality of the receiving low order streams. However, the extent of this impact is rarely monitored. We installed discharge and water quality measurements at the outlet of two small, neighbouring headwater catchments, one that includes a village, a WWTP, and two CSOs, while the other is predominantly influenced by agricultural activities. We also deployed electrical conductivity (EC) loggers at the CSOs to accurately detect discharge times. Discharge from the WWTP and CSOs led to higher peak flows and runoff coefficients during events. Less dilution of EC and increasing ammonium-N (NH_4_ − N) and ortho-phosphorus (oPO_4_ − P) concentrations indicate a significant contribution of poorly treated wastewater from the WWTP. During CSO events, water volumes and nutrient loads were clearly elevated, although concentrations were diluted, except for nitrite-N (NO_2_ − N) and particulate phosphorus (PP). Baseflow nitrate-N (NO_3_ − N) concentrations were diluted by the WWTP effluent, which led to considerably lower concentrations compared to the more agriculturally influenced stream. Concentrations of oPO_4_ − P, NH_4_ − N, and NO_2_ − N, which are most likely to originate from the WWTP, vary throughout the year but are always elevated. Our study shows the major and variable impact rural settlements can have on stream hydrology and water quality. Point sources should be monitored more closely to better understand the interaction of natural catchment responses and effects caused by sanitary infrastructure.

**Supplementary Information:**

The online version contains supplementary material available at 10.1007/s10661-023-12051-6.

## Introduction

Reaching at least a “good status” of rivers, lakes, coastal waters, and groundwaters until 2027 is a key requirement of the EU Water Framework Directive (WFD) (European Union, 2000). An integrated approach, taking into account point sources, such as discharges from wastewater treatment plants (WWTPs), as well as diffuse sources from agricultural activities, is a key aspect of the WFD. Within the Urban Wastewater Treatment Directive (European Union, 1991), which specifies and complements the WFD, requirements for urban waste waters are formulated. However, WWTPs, as well as sewer systems (SS), and their associated combined sewer overflows (CSOs), only have to meet these requirements if they are associated with urban agglomerations of a specific size: The German law, for example, formulates no regulations for nutrient concentrations in effluents of WWTPs smaller than 5000 population equivalents (PE) (AbwV, 1997). Many of these WWTPs therefore lack a phosphorus elimination, a denitrification, or even a nitrification, with some facilities only being equipped with biological and mechanical treatment (HMUKLV, 2021). In the federal state Hesse (Germany) alone, there are 317 WWTPs with a PE of less than 2000, of which only 63 are equipped with all four treatment steps (HMUKLV, 2021). In most cases, the streams receiving these effluents are of first or second order. Consequently, this means the dilution of the effluent is limited (Wade et al., 2012). Especially during low flow situations, cleaned wastewater was found to be a major tributary to streams, delivering high concentrations and/or loads of nutrients, sediments, and organic pollutants (Müller et al., 2018; Jarvie et al., 2004; Phillips et al., 2012; David et al., 2013; Halliday et al., 2015). Additionally, small WWTPs are often less resistant regarding disruptive factors such as increased inflow, which can lead to fluctuating outflow concentrations (Schlüsener und Bester, 2008; Phillips et al., 2012; Neal et al., 2008). This might endanger aquatic ecosystems of small rivers, since they are sensitive to changing external factors (Matzinger et al., 2012).

During storm events, CSOs and WWTPs can increase the natural flow and reduce the response time of the whole catchment (Schwientek et al., 2013; Halliday et al., 2014; Bowes et al., 2015). The storage capacity of SSs, and thus the likeliness of discharge from CSOs, is depending on its size and previous meteorological conditions (Gallé et al., 2018). The contributions of wastewater from this source therefore changes depending on location and season (Schwientek et al., 2013). The water quality of CSO discharges is an additional stress factor for streams and was found to be variable depending on previous weather conditions as well (Deffontis et al., 2013). Especially critical are xenobiotics and ammonium, of which elevated concentrations already have been detected within streams receiving water from CSOs (Gallé et al., 2018; Lawler et al., 2006). However, instead of being included into the monitoring scheme, the influence of point sources is frequently just estimated: Wastewater is often assumed to have constant water quality throughout events, while mean annual loads are approximated based on regular baseflow grab samples (Müller et al., 2018; Ehrhardt et al., 2019; Halliday et al., 2014; Kyllmar et al., 2014). Samples taken on an event basis are rarely available.

Recent studies discuss urban areas as a small part of medium to bigger sized catchments (Bowes et al., 2015; Zimmer et al., 2019; Halliday et al., 2014; Baker et al., 2003). This approach makes it difficult to distinguish between urban, agricultural, and natural influences due to the integration of different signals (Wade et al., 2012; Halliday et al., 2015). It also makes it impossible to quantify processes in the upper reaches of the catchments, although these are highly relevant further downstream (Burns et al., 2009; Alexander et al., 2000). Studies which take sanitary infrastructure more detailed into account often set a focus on the discharge from WWTPs only, missing out CSOs (Ehrhardt et al., 2019; Bowes et al., 2015). Other studies focus on bigger cities and highly urbanised catchments (Lawler et al., 2006; Carter et al., 2003; Duncan et al., 2017; Silva et al., 2002; Vaughan et al., 2017), where the direct identification of sources is difficult, due to the complex and dense network of SSs, storm sewers, and CSOs. Some studies therefore monitor only small river sections downstream of point sources (Baker et al., 2003; Gallé et al., 2018; David et al., 2013). The monitoring of single CSOs, like performed by Deffontis et al. (2013), however, does not allow conclusions concerning stream water quantity and quality. Despite the potential of better separating the different sources and processes in smaller catchments, a special focus on the impact of (smaller) SSs and WWTPs on water quantity and quality in low order catchments is rarely set (Müller et al., 2018; Wade et al., 2012).

To fill this research gap, we installed discharge and water quality measurements in two neighboured headwater catchments, one of them includes a village, CSOs, and a WWTP. We wanted to answer the question in which ways sanitary infrastructure alternates the dynamics of water quantity and quality in the upper reaches of otherwise natural and agriculturally influenced catchments.

## Materials and methods

### Study sites

**Fig. 1:**
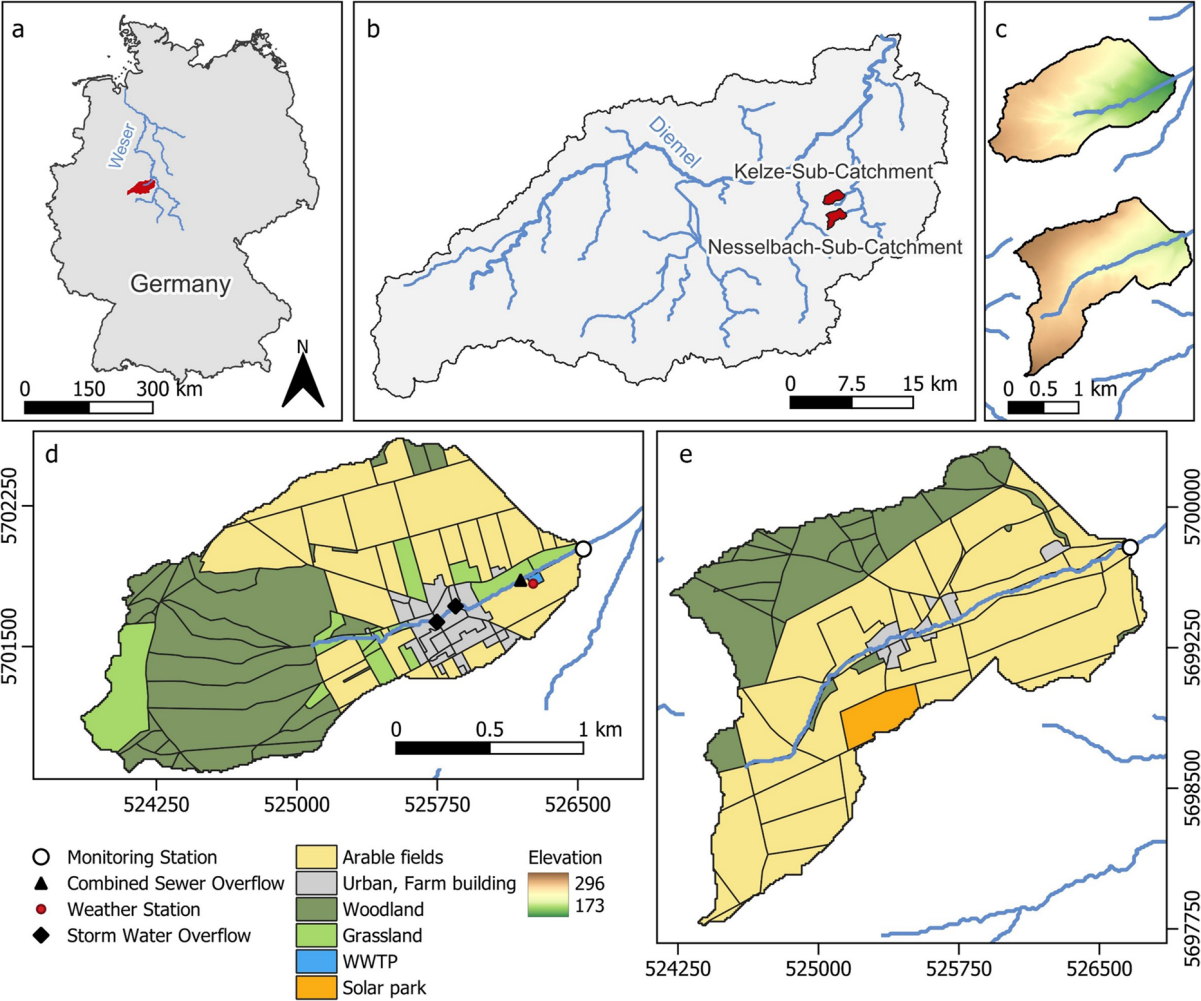
Location of the research area in Germany **(a)** and within the Diemel catchment **(b)**, elevation map **(c)**, land use and monitoring site A (Kelze) **(d)**, and site B (Nesselbach) **(e)**; WWTP, wastewater treatment plant

The two investigated low mountain range headwater catchments are located next to each other in the north of Hesse, Germany. Major characteristics are summarised in Table 1. The gauged catchment of the Kelze stream (site A) covers an area of 2.77 km^2^. The gauged area of the Nesselbach (site B) stream is slightly bigger, draining an area of 2.93 km^2^. Compared to other studies (Bowes et al., 2015; Schwientek et al., 2013), we chose relatively small headwater catchments to limit complexity and to better separate between different sources (Jarvie et al., 2000). Both catchments are tributaries to the Esse River within the Weser River catchment (Fig. 1). Catchment geology on both sites is dominated by low Muschelkalk karst aquifers, which are overlain by brown earth soils with depth ranging from 0.5 to more than 2 m. During the 1-year monitoring period from February 2021 until January 2022, total precipitation summed up to 580 mm. During July and August, heavy rainfall occurred, exceeding the long-term mean for these months, followed by a dry autumn and winter. The mean yearly temperature was 9.9 °C, with snowfall and freezing temperatures until mid-February.

**Table 1:** Catchment characteristics. *PE*, population equivalent; *WWTP*, wastewater treatment plant; *CSOs*, combined sewer overflows

	Kelze (site A)	Nesselbach (site B)
Area [km^2^]	2.77	2.93
Landuse [%]		
Urban	6.8	2.2
Agriculture	43.1	69.6
Forest	39.1	25.4
Min elevation [m a. s. l]	173.3	202.3
Max elevation [m a. s. l]	282.4	296.1
Mean slope [%]	7.5 ± 5.5	6.7 ± 4.3
Geology	Red Sandstone and Low Muschelkalk	
Soils	Pseudogley, Pelosol-Brown earth, Luvisol, Cambisol	
Urban influence		
Population	279	60
PE of WWTP	350	-
CSOs	2	-

Agricultural land use, which included mostly maize and wheat, is more predominant on site B (69.6%) compared to site A (43.1%). During spring and autumn, the application of fertilizer was observed. Within site B, a few farms can be found, accounting for 2.2% of the catchment area. Although urban areas are also comparatively small on site A (6.8%), there is a village and a WWTP with a PE of 350 located in the catchment. Most of the village is drained by a combined SS, and only a small development area has a separated SS for stormwater, which directly discharges runoff into the stream. Drinking water is received from a different catchment close by. Thus, water consumed by the inhabitants is additional water in the catchment water balance. The WWTP consists of four ponds, which are connected in a row. The first two ponds are aerated (biological treatment); the other two are clarification ponds. Water flow is only controlled by gravity and the size of the pipes. No pumps or valves are installed to control discharge during events. To protect the WWTP from spilling, there are two CSOs. When water reaches a specific level within the SS, it spills over a weir edge and gets directly discharged into the stream. A second overflow is directly located before the first pond.

### Measurement setup

A weather station (Thies Clima, Adolf Thiess GmbH & Co. KG) was installed on the area of the WWTP, measuring precipitation, temperature, windspeed, solar radiation, vapour air, and humidity in a 5-min interval. Since the catchments are located only 2 km apart from each other, the data was assumed to be valid for both sites.

On site A, we installed a Thomson weir and a pressure probe (HOBO U20L-04 S/N, Onset Computer Corporation) for monitoring water levels. Discharge was calculated by the Thomson weir formular (Nützmann und Moser, 2016). The Nesselbach (site B) was equipped with a magnetic inductive discharge measurement (PCM Pro, NIVUS GmbH). We performed salt tracer experiments during different discharge stages at both sites to correct and validate the calculated discharges. To minimise uncertainties due to erosion and sediment accumulation within the streambed (Harmel et al., 2005), we removed sediments on a regular basis and after bigger storm events. Multiparameter spectrometer probes (spectro::lyser V3, s::can GmbH) were installed on both sites to measure temperature, nitrate-N (NO_3_ − N) concentrations, and turbidity, while electrical conductivity (EC) was measured by HOBO probes (HOBO U24-001, Onset Computer Corporation). Intervals for measurements were set to 5 min. Gaps in the data set are mainly due to occasional system malfunction. This affects 16.4% and 10.2% of the EC measurements for sites A and B respectively and 16.8% of the discharge data on site B. For monitoring the CSOs, we installed modified Onset HOBO Pendant waterproof temperature and light data logger (UA-002-64, Onset Computer Corporation) to measure EC (Chapin et al., 2014; Lieder et al., 2017) in each of the CSOs. As soon as the sewer overflows start to discharge, EC increases significantly. Grab samples were taken on a bi-monthly to monthly basis to validate the probe measurements and to additionally determine ammonium-N (NH_4_ − N), nitrite-N (NO_2_ − N), total phosphorus (P_tot_), and ortho-phosphorus-P (oPO_4_ − P). To also monitor these nutrients during single events, we installed automatic samplers (ISCO 6712 (site A) and ISCO 3700 (site B)). The samplers were controlled by conductivity sensors, which initiated and stopped sampling depending on whether the sensors were in contact with stream water or not, leading to different number of samples for each event. The sampling frequency was reduced step by step from every 15 min for the first samples to hourly time steps for the later samples. The samples were taken to the Laboratory for Urban Water Management and Water Quality (University of Kassel) within 12 h. All samples except the ones for analysing P_tot_ were filtered through 0.45-μm fiberglass filters into HDPE bottles right away. Nutrients were either measured immediately or kept frozen for later analysis. NO_3_ − N was measured using an ion chromatograph (791 Compact IC, Metrohm AG). All other parameters were measured with an UV-VIS spectrometer (Agilent Cary 100 UV-Vis, Agilent Technologies). Methods, standards, and uncertainty ranges are summarised in Tab. 1S in the Supplementary Information. We collected 261 event (during 17 events) and 27 baseflow samples on site A and 214 event (during 18 events) and 16 baseflow samples, on site B, which complemented the high-frequency data. Further details are summarised in Tab. 2S in the Supplementary Information.

In contrast to other studies, we installed our river measurements on site A shortly after the point sources. Longitudinal dispersion and mineralisation processes are therefore expected to be low (Gallé et al., 2018), meaning that we could capture the “original” signal of point source water contaminants. This is especially important for ammonium, which is subject to nitrification when reaching oxidised streams (Brion und Billen, 2000).

### Data analysis

#### Data handling and pre-processing

All statistical analyses were performed using the statistical language R (R Core Team, 2022). In a first step, data outliers, such as sudden drops, rises, or zero values, were deleted in the high-frequency probe data (Aguinis et al., 2013). Data gaps of less than 3 h were linearly interpolated. A running median filter from the R package *robfilter* (Fried et al., 2019) was used to reduce data noise (window width = 7). During baseflow, the noise of the discharge data on site B was very strong. We therefore extended the window width during baseflow to 24, which is equal to 2 h.

Salt tracer experiments showed that discharge measured by the probe on site B was always overestimated. However, the Pearson correlation coefficient (*R*) between instantaneous discharge and discharge calculated from salt tracer experiments showed a strong linear relationship (*R*=0.998), with no seasonal changes. The discharge data was therefore corrected with a linear regression. To assess the agreement between in situ and laboratory measurements, we calculated the Pearson correlation (*R*) coefficient and the root mean square error (RMSE). We divided the year into four seasons to identify seasonal variation in the data. Statistics were calculated for winter (JFM = January, February, March), spring (AMJ = April, May, June), summer (JAS = July, August, September), and autumn (OND = October, November, December).

#### Baseflow separation and event definition

To calculate baseflow, we used the Lyne-Hollick three pass recursive digital filter (Lyne und Hollick, 1979):

1 $${q}_f(i)=\left\{\begin{array}{c}\alpha {q}_f\left(i-1\right)+\frac{\left(1+\alpha \right)}{2}\left[q(i)-q\left(i-1\right)\right]\kern1em \textrm{for}\ {q}_f(i)>0\\ {}0\kern16em \textrm{otherwise}\ \end{array}\right.$$

2 $${q}_b(i)=q(i)-{q}_f(i)$$

where *q*_*f*_(*i*) and *q*_*b*_(*i*) are the quickflow and baseflow responses at timestep *i*, and *q*(*i*) is the original streamflow. The filter parameter *α* was set to 0.925 (Murphy et al., 2009; Nathan und McMahon, 1990). The baseflow index (BFI) was then calculated as the ratio of baseflow to streamflow. Although physical processes are not taken into account, this method for baseflow separation was found to be particularly useful to characterise differences between catchments, due to its objectivity and consistent manner (Ladson et al., 2013). Ladson et al. (2013) and Murphy et al. (2009) suggest increasing the number of passes to 9 times, when using the filter on higher resolution data. We followed this suggestion. A storm event was then defined by discharge exceeding the baseflow of more than 20% with an increase of discharge during the event of at least 4 L s^−1^. For events, we calculated the event-based runoff coefficient (RC) as the ratio of total event discharge to total precipitation. We also estimated the maximum percentual change in discharge, EC and NO_3_ − N concentration, by calculating the ratio between the maximum or minimum measured value during the event and at the beginning of the event.

#### Load calculation

It was not possible to collect samples during every storm event. Therefore, the application of linear interpolation to our data set for the calculation of nutrient loads would introduce a biased error (Birgand et al., 2010), as it would ignore increasing or decreasing concentrations during unmonitored events. We therefore decided to follow a flow-weight approach to calculate nutrient loads *P*_*L*_ [kg] (Moatar und Meybeck, 2005):

2 $${P}_L=K\frac{\sum_{i=1}^n{c}_i{Q}_i}{\sum_{i=1}^n{Q}_i}\overline{Q}$$

*K* is a conversion factor which accounts for the period of load estimation and units, and $\overline{Q}$ is the mean discharge [L s^−1^]. *C*_*i*_ and *Q*_*i*_ are the sample concentration [mg L^−1^] and the corresponding discharge [L s^−1^] at timestep *i*. This method was found to be robust for estimating load fluxes based on low-frequency pollution data. Using monthly to bi-monthly data, the uncertainty is expected to range between 6.5% for NO_3_ and 8.8% for oPO_4_ (Moatar und Meybeck, 2005). We took uncertainties of discharge and concentration measurements into account to calculate upper and lower uncertainties for load export. To compare total inorganic nitrogen (N_tot_) between both sites, we defined N_tot_ as the sum of NO_3_ − N, NO_2_ − N, and NH_4_ − N. Particulate phosphorus (PP) was calculated as the difference of P_tot_ and oPO_4_ − P.

## Results and discussion

### Uncertainty in the in situ data

In situ and laboratory measurements of NO_3_ − N and EC were in good agreement, with only a few outliers (Supporting Information Fig. 3S) and with correlation coefficients > 0.96 (*p* < 0.001). For discharge and EC, uncertainties defined by the probe specifications were found to be appropriate. RMSEs for discharge calculation vs. calculation based on the salt tracer experiments was 1.4 L s^−1^ on site B and 0.7 L s^−1^ on site A. The RMSE between laboratory and in situ EC measurements were 16.0 μS cm^−1^ on site A and 18.9 μS cm^−1^ on site B. Uncertainty for NO_3_ − N measurements, as defined by the manufacturer’s specification, was found to be too optimistic. For site A, we calculated a RMSE of 0.5 mg NO_3_ − N L^−1^. To include most of the data points into the uncertainty bounds, we estimated ±0.68 mg NO_3_ − N L^−1^ to be a fitting uncertainty range for nitrate –N measurements. On site B, NO_3_ − N uncertainties were found to be wider (RMSE=0.7 mg L^−1^) with estimated bounds of ±1.1 mg NO_3_ − N L^−1^.

### Baseflow conditions

**Fig. 2:**
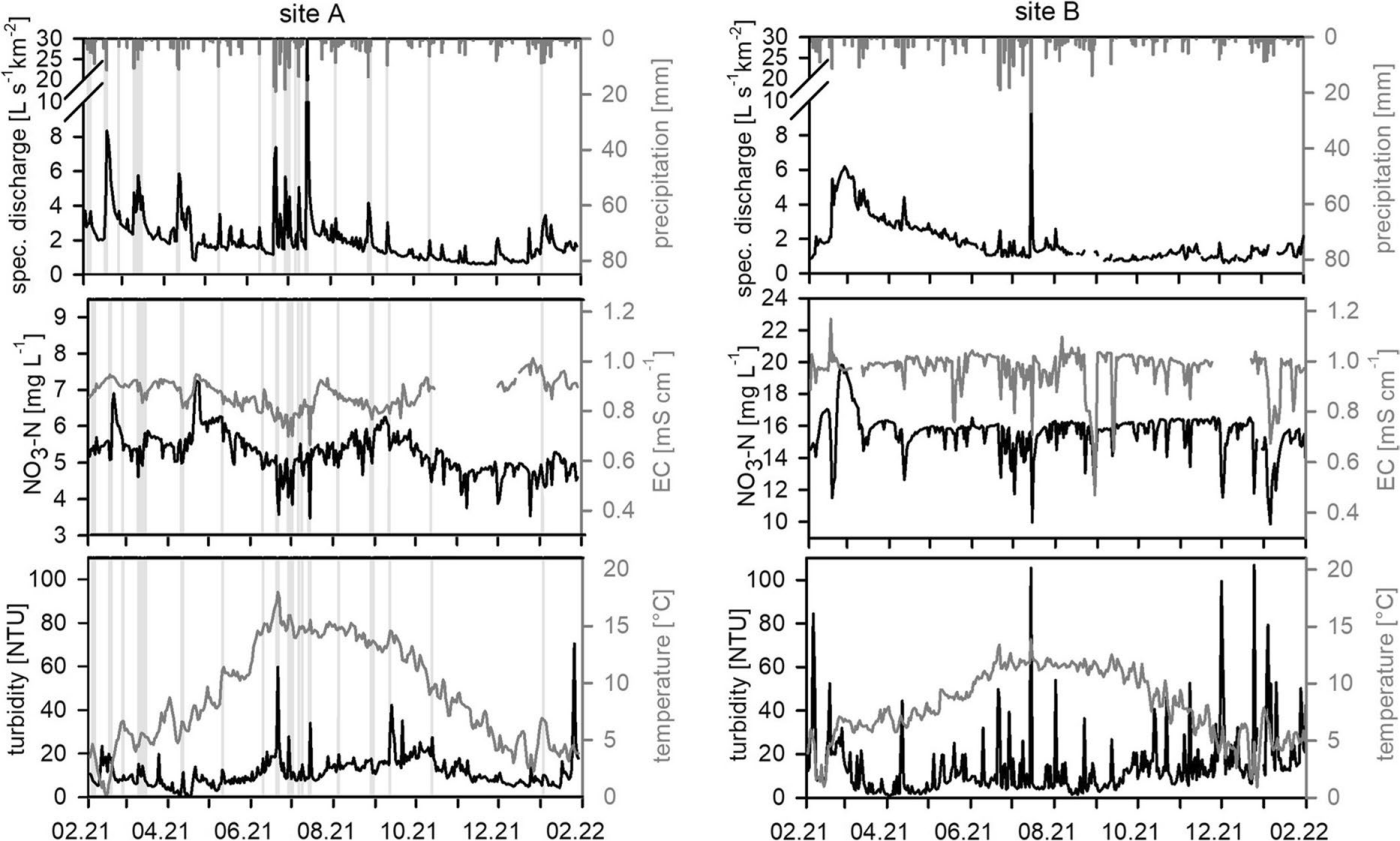
Daily discharge, NO_3_ − N concentration, EC, turbidity, and temperature on site A (left) and site B (right); the grey vertical lines on site A show CSO events; the scaling and ranges of the NO_3_ − N axes differ between both sites; *EC*, electrical conductivity; *CSO*, combined sewer overflow

**Fig. 3:**
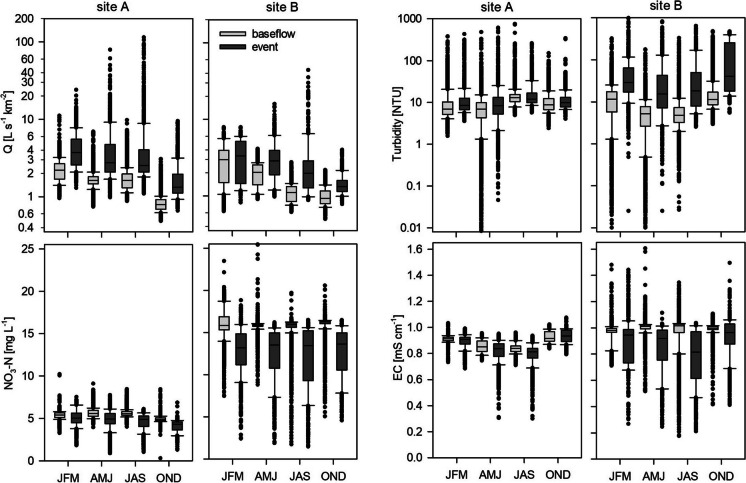
Distribution of high-frequency data (15-min average) separated for seasons and baseflow and event conditions; all differences of the mean of the distribution of baseflow and event flow data are significant (Mann-Whitney *U* test, *p* < 0.001); *JFM*, January, February, March; *AMJ*, April, May, June; *JAS*, July, August, September; *OND*, October, November, December; *EC*, electrical conductivity

On site B, discharge decreased throughout the year, with high baseflow during winter (JFM), when groundwater was recharged, followed by a slowly release during spring (Fig. 2 and Fig. 3). The heavy rainfalls during summer (JAS) did not result in an increase of baseflow (Fig. 2). The event water rather seems to be stored and released constantly throughout the dry period in OND, leading to a higher baseflow (mean: 1.0 L s^−1^km^−2^) compared to site A (mean 0.8 L s^−1^km^−2^). The steady EC measurements support this assumption, as they indicate a constant water source.

On site A, 50% of baseflow discharge fell within a relatively narrow range within the boxplots (Fig. 3), indicating stable discharge conditions and a prominent baseflow, with only slightly varying seasonal NO_3_ − N concentrations. The continuous discharges from the WWTP might contribute to that situation (Schwientek et al., 2013). But it is also likely that the catchment additionally formed NO_3_ − N legacy stores within the soil and the groundwater, which is typical for managed catchments with constant N− input from fertilizer, atmospheric deposition, or sewage leaks (Basu et al., 2010; Ehrhardt et al., 2019; Burns et al., 2019; Duncan et al., 2017). Due to the high share of agricultural fields (Table 1), the latter explanation is also more likely to be the reason for the constant and very high NO_3_ − N concentrations at site B. The yearly mean concentration of 15.3 mg L^−1^ exceeds the threshold of 11.3 mg NO_3_ − N L^−1^ of the EU nitrate directive (91/676/EWG, 1991) and was significantly higher compared to site A (Mann-Whitney *U* test; *p* < 0.001), where the average concentration was 5.3 mg L^−1^.

During early spring, a “hot moment” for nitrate export occurred, with increased baseflow discharges and NO_3_ − N concentrations reaching maximum values of 20 mg L^−1^ on site B. In Germany, farmers are allowed to spread fertilizer from the 1^st^ of February, which might have been the case at our study sites. In 2021, there was significant snowfall right after this deadline. Organic nitrogen and ammonium can get mineralised underneath the snowpack, resulting in a “hot moment” for nitrate export during spring, when the snow is melting and nitrate is transported to the river via lateral flow (Vidon et al., 2010). This effect was less pronounced on site A, although NO_3_ − N concentrations and baseflow were elevated as well, reaching 6.8 mg L^−1^. The village might have led to a damping of the discharge curve: Runoff from snowmelt within the village gets transported to the WWTP via the SS, instead of reaching the stream immediately. Site A also has less agricultural fields and more grassland directly adjoining the Kelze stream, resulting in longer transport times and thus more possibilities for nutrient retention (Casal et al., 2019).

**Fig. 4:**
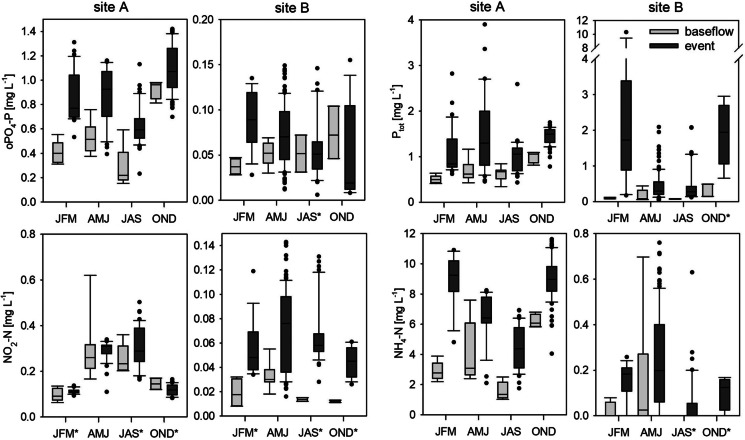
Distribution of low-frequency data from grab samples and automatic probe samplers, separated for season and baseflow and event conditions; * indicates non-significant differences of the mean of the distribution of baseflow and event flow data for the specific substance and season, all other differences are significant (Mann-Whitney *U* test, *p* <= 0.05–0.001); axes division differ between both sites; *JFM*, January, February, March; *AMJ*, April, May, June; *JAS*, July, August, September; *OND*, October, November, December

During several days in April, construction works at the WWTP led to reduced outflow. Stream discharge decreased to approximately 1 L s^−1^ km^−2^. During this period, NO_3_ − N concentrations increased to more than 7.0 mg L^−1^, exceeding the concentrations from the snowmelt period. Groundwater and thus stream NO_3_ − N concentrations are probably high but are diluted by the effluent of the WWTP under normal conditions. In many studies, the opposite behaviour was found, with WWTPs enhancing NO_3_ − N concentrations during baseflow (Burns et al., 2019; Wade et al., 2012). Turbidity, on the other hand, reached its minimum values (lower outliers Fig. 4). This suggests that otherwise the WWTP is a constant source for suspended solids, e.g. decaying organic matter or biological solids, such as heterotrophic and nitrifying bacteria (Brion und Billen, 2000). This leads to constant high turbidity levels, even during the summer months, when erosion is expected to be low due to vegetation, as it was observed on site B.

The highest NO_3_ − N concentrations (7 mg L^−1^) on site A, however, were measured when sudden increases of discharge occurred, which were not related to any precipitation. The unknown source released high amounts of water, introducing short-term increases of discharge of up to 30 L s^−1^. This happened 35 times throughout the year. These events explain the upper outliers in the boxplots in Fig. 3 for the baseflow discharge and NO_3_ − N components. During June, July, and August, the automatic sampler captured some samples. NH_4_ − N spiked during June (7.6 mg L^−1^), while it stayed constant during July and August. Discharge of untreated wastewater, for which high NH_4_ − N concentrations would be typical, is very unlikely, because the increase of NO_3_ − N concentrations exceeded the concentrations typically measured within the WWTP effluent (not published). Groundwater can also be ruled out as a source, since NH_4_ − N concentrations are typically lower in groundwater. On site B, four short-term NO_3_ − N spikes occurred as well, but they were not connected to any change in discharge, but to a rise in EC. The spikes could, for example, originate from urban or agricultural activities, which are likely for such areas (Burns et al., 2019; Bende-Michl et al., 2013).

On site B, NH_4_ − N and NO_2_ − N were mainly detected in samples during winter and spring (JFM, AMJ, Fig. 4). Phosphorus seems to be exported predominantly bound to particles, indicated by the much higher concentration of P_tot_ compared to oPO_4_ − P. PP was particularly relevant during spring (AMJ) and autumn (OND). Less vegetation, snow melt, and the harvesting of fields may have led to the mobilisation of particles during baseflow (Bowes et al., 2015).

On site A, concentrations of NH_4_ − N and NO_2_ − N were significantly higher (Mann-Whitney *U* test, *p* < 0.001), exceeding water quality standards for drinking water and groundwater in Germany of 0.39 mg L^−1^ and 0.15 mg L^−1^, respectively (TrinkwV, 2016; GrvW, 2010). P_tot_ concentrations were only slightly higher than oPO_4_ − P concentrations, indicating that the majority of phosphorus is transported as oPO_4_ − P. oPO_4_ − P median concentrations during baseflow ranged around 0.5 mg L^−1^. This is higher than the threshold of 0.1 mg P L^−1^, which was defined as a trigger point for excessive algal growth by Jarvie et al. (2004). Elevated concentrations of these N− and P− fractions are typically found in WWTP outflow (Bowes et al., 2015; Halliday et al., 2015; Wade et al., 2012). The WWTP on site A only has a biological treatment, which favours a decrease in organic carbon, but still leaves high NH_4_ − N and oPO_4_ − P concentrations in the effluent.

Baseflow EC, NO_3_ − N, NH_4_ − N, and oPO_4_ − P concentrations decreased after the heavy rainfall period in July (Fig. 2). The storage of event water within the SS and the WWTP and a delayed release might be the reason for the dilution effects even after the events. Median P_tot_ concentrations slightly rose due to a higher share of PP during this period. Particle-bound phosphorus from the stream bed was probably mobilised (Bowes et al., 2012), which is also indicated by the simultaneously rising turbidity.

Maximum baseflow concentrations of 7 mg L^−1^ and 0.95 mg L^−1^ for NH_4_ − N and oPO_4_ − P, respectively, were reached during the comparatively dry autumn (OND). Similar behaviour was found by Bowes et al. (2015) and Wade et al. (2012), who suspected less dilution of WWTP outflow during low flow periods. However, the outflow of the WWTP itself might also vary. Neal et al. (2008), e.g., measured phosphorus spikes in WWTP effluents, which were not correlated to discharge. Microbiological organisms are accustomed to specific milieus (Cho et al., 2014). Temperature and pH values, but also residence times, organic carbon content, and substance concentrations influence the efficiency of nutrient elimination (Cho et al., 2014; Limpiyakorn et al., 2005; Bowes et al., 2012). The water use of the inhabitants, infiltration, and inflow into the sewer system, but also hydrological conditions, such as the intensity, duration, and quantity of the last precipitation event, additionally influence dilution and inflow quantities. Dry periods can support a worst-case scenario, where NH_4_ − N and oPO_4_ − P are still high in the effluent, while stream discharge is very low. Maximum NO_2_ − N concentrations occurred during spring and summer, reaching values up to 0.78 mg L^−1^, and thus exceeding event concentrations, while winter and autumn baseflow concentrations were below 0.2 mg L^−1^. NO_2_ − N, which is an intermediate product during nitrification, points towards active transformation processes within the stream, especially during the warmer periods. Nitrifying bacteria can be found in the effluent of WWTPs (Cébron et al., 2003; Brion und Billen, 2000). Thus, NH_4_ might be transformed to NO_2_ and NO_3_, as soon as it reaches the oxygen-rich stream, and therefore be an indirect source for nitrate in the downstream reaches. During colder temperatures, nitrification and denitrification processes slow down (Halliday et al., 2015), which explains the lower NO_2_ − N concentrations during autumn and winter.

### Event flow

Specific discharge and water quality characterisitcs are summarized in Table 2. Event discharge was much more pronounced on site A compared to site B, with steep increases and higher peaks, represented by the higher upper whiskers and outliers (Fig. 3). On average, discharge increase was 703% compared to pre-event conditions, with mean event discharges of 4.1 L km^−2^ s^−1^. After events, a gentle recession could be observed. During rainy periods, discharge did not reach baseflow before the next event started, leading to a mean event duration of 21.4 h (median: 12.3 h) and several peaks within one event. Some events resulted in an increase of discharge on site A, but did not have any significant effect at site B.

**Table 2:** Discharge and water quality characteristics calculated from high-resolution data. *BF*, baseflow; *BF*, baseflow index; *RC*, runoff coefficient; *EC*, electrical conductivity

	Site A	Site B
Discharge characteristics		
Mean spec. discharge [L km^−2^ s^−1^]	2.1±2.8	1.9±1.5
No. of events [-]	74	73
Max discharge [L km^−2^ s^−1^]	130.7	21.1
Mean event flow [L km^−2^ s^−1^]	4.1±6.0	2.8±2.8
Mean rise of discharge [%]	671	262
Mean spec. BF [L km^−2^ s^−1^]	1.4	1.5
Mean BFI [-]	0.80	0.81
Mean event BFI [-]	0.55	0.68
Mean RC [-]	0.023	0.011
Water quality characteristics		
Max NO_3_ − N [mg L^−1^]	10.5	32.9
Min NO_3_ − N [mg L^−1^]	3.7	6.4
Mean NO_3_ – N [mg L^−1^]	5.3±0.7	15.3±1.7
Mean decrease of NO_3_ − N [%]	41	50
Mean decrease of EC [%]	17	41

Mean RC on site A was 0.023. Due to different methodologies concerning baseflow separation, this value is difficult to compare with literature (Blume et al., 2007). However, compared to site B, the differences become very clear, as the mean RC there was 0.011. Mean discharge rise was 286%, with mean event flows of 2.8 L km^−2^ s^−1^. Discharge showed a steep recession and returned fast back to baseflow, with a shorter mean event time of 13.6 h (median: 10.3 h). The sealed area and the SS might be responsible for the more frequent and higher peaks at site A, as they tend to decrease the transit times of catchments (Schwientek et al., 2013). At the end of events, the WWTP as well as the SS might have worked as a storage, which would explain the generally longer event durations and the slow decrease back to baseflow, as the equilibrium within the WWTP regenerates. Especially during the series of events in June and July 2021, this difference becomes quite clear. On site A, the prolonged rainfalls resulted in several peaks (and CSO events) while on site B the catchment reaction is less pronounced. Only the heavy rainfall event in July, where in total 55 mm precipitation and maximum rainfall intensities of 10 mm h^−1^ occurred, resulted in a steep increase of discharge on site B, exceeding all other events.

Considering discharge together with EC and water quality parameters allows some first conclusions regarding runoff generation in the catchments. On site B, the mean decrease of EC during all events was 42%. Compared to site A, the boxplots for EC were more distributed, outliers were lower, and median event values differed significantly from baseflow values. NO_3_ − N was diluted during events as well. Both parameters are negatively correlated with the BFI (Table 3). At the end of events, when discharge already got back to baseflow, NO_3_ − N as well as EC did not reach their initial concentrations, which is represented by the lower outliers at baseflow conditions. Taking this information into account, two discharge components can be identified at site B: at the beginning of an event, a big share of rainwater directly reached the stream via fast runoff and shallow lateral flow. Later on, subsurface flow still gets diluted by infiltrating rain, but gets slowly enriched again, by an NO_3_ − N rich groundwater component (Bende-Michl et al., 2013). The fast runoff also mobilised sediments, and thus PP, as it is indicated by the strong correlation between turbidity and P_tot_ (*R* = 0.86), while only a small share of phosphorus was present as oPO_4_ − P. During autumn (OND) and winter (JFM), turbidity and P_tot_ concentrations reached their maximum (P_tot_ up to 10 mg L^−1^). Concentrated overland flow through riparian buffers can lead to “hot moments” for P− export, which was also found in other agricultural catchments (Bende-Michl et al., 2013).

**Table 3:**
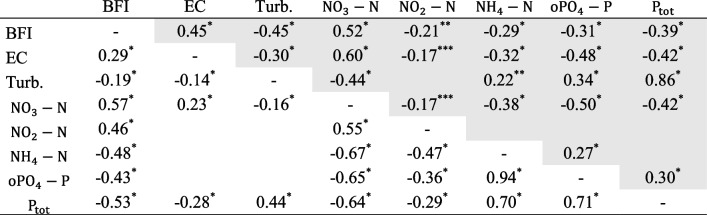
Significant parameter correlations of nutrient concentrations, turbidity, EC, and BFI for site A (lower left side; white background) and site B (upper right side, grey background); *BFI*, baseflow index, *EC*, electrical conductivity, *Turb.*, turbidity. ***p<0.05, **p < 0.01, *p < 0.001

The high event discharge on site A suggests that quickflow and thus rainwater seem to contribute a big share of runoff. However, the relationship between BFI and EC is less pronounced (*R* = 0.29). Also, median event EC is not much lower compared to baseflow EC (Fig. 3), with a mean reduction of only 18% during events. The relationship between NO_3_ − N dilution and increase of discharge on the other hand lies within a similar range as observed on site B. A possible explanation would be that additionally to rainwater reaching the stream via runoff or lateral flow, also wastewater, which has higher EC and NO_3_ − N concentrations compared to precipitation, is released from the WWTP. Due to the combined SS, a big share of the runoff within the village is collected and directly transported to the WWTP. The SS is quite small, with a short distance to the WWTP, and thus fast water transit times. Additional water reaching the WWTP might interrupt the cleaning processes, leading to an increase of NH_4_ − N and oPO_4_ − P concentrations, which are strongly positively correlated (*R* = 0.94), while they are negative correlated with the BFI. Discharges from WWTPs can vary strongly during events (Phillips et al., 2012; Neal et al., 2008). Varying removal efficiencies between dry and wet weather have already been confirmed for pharmaceuticals (Fono und Sedlak, 2005). Schlüsener und Bester (2008) even found a collapse of the biological treatment of a small WWTP (32 000 PE) during heavy rainfall events. Bowes et al. (2012), on the other hand, made a slightly different observation with P in WWTP effluent from a treatment plant including phosphorus elimination. They found P concentrations in the effluent not being correlated with short-term event peaks, but they found a long-lasting increase of P after a longer rainfall period, indicating that P removal was not as efficient in times with a high volumetric throughput. This shows the strong variety of small WWTPs, while bigger plants were found to be more robust against variation in discharge (Schlüsener und Bester, 2008).

During events, PP became also more present at site A. However, many fields close to the Kelze stream are partly used as grassland and pastures, decreasing chances for a particle-bound overland transport of phosphorus. The WWTP could be the responsible source as well, as the adsorption of P from WWTPs onto stream sediments, followed by a sedimentation and a mobilisation during storm events, is a commonly observed process (Bowes et al., 2015; Wade et al., 2012; Bowes et al., 2012).

### Nutrient loads

On site A, a discharge of 23.3 · 10^3^ m^3^ km^−2^, which accounted for 36% of total yearly water exports, left the catchment during events in only 18% of the time. On site B, where event discharge was present during 12% of the time, total event water accounted for 13% of water and summed up to 8.8 · 10^3^ m^3^ km^−2^.

**Fig. 5:**
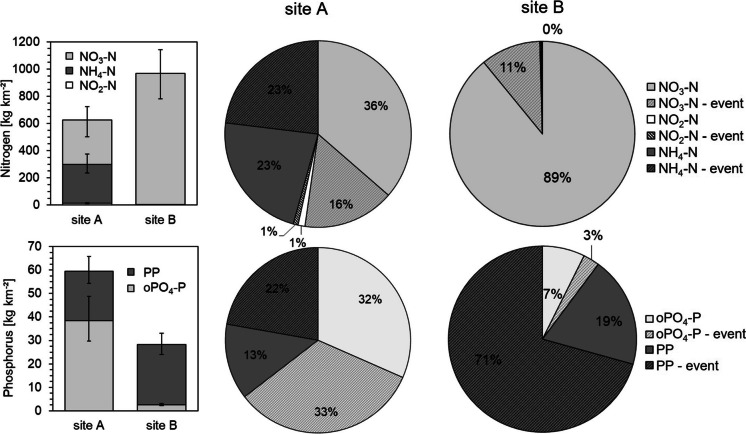
Left side: total exported nutrient fractions during the monitoring year; right side: fractions of nitrogen and phosphorus loads on both sites exported during event and during baseflow

The total N− loads during the monitoring year were in a similar range as the loads calculated by others (Outram et al., 2016; Ehrhardt et al., 2019), reaching 625 kg N km^−2^ on site A (Fig. 5) and 909 kg N km^−2^ on site B. On site B, total riverine N− load was mostly present as NO_3_ − N (905 kg km^−2^), while NH_4_ − N and NO_2_ − N accounted for less than 1%. In contrast, NH_4_ − N accounted for 46% (286.9 kg km^−2^) and NO_2_ − N for 2% (12.7 kg km^−2^) on site A, while only 325 kg km^−2^ were exported as NO_3_ − N. The contributions of the different nitrogen species are very dependent on the treatment steps within the WWTPs. Halliday et al. (2014), for example, found NO_3_ − N being the predominant nitrogen species in the effluent and the river. The WWTP at their study site probably included a nitrification.

Total event derived NO_3_ − N loads were in a similar range on both sites (99.3 kg km^−2^ on site A, 95.2 kg km^−2^ on site B), which is surprising, as the NO_3_ − N concentrations on site B were generally higher. Taking all N− fractions on site A into account, event-export accounted for 40% of total nitrogen export (249 kg N km^−2^ a^−1^) and exceeds the event export on site B (98.1 kg km^−2^ a-1; 11%) by more than double.

While events seem less relevant for nitrogen riverine loads on site B, this picture turns when discussing P_tot_ loads (25 kg km^−2^): 71% of P_tot_ the catchment as PP during events (17.6 kg km^−2^). Total event PP riverine load on site A was slightly less (13.2 kg km^−2^). On site A, oPO_4_ − P is the more dominant P− fraction (65%). Similar as observed for NH_4_ − N, half of the oPO_4_ − P loads are exported during events (19.6 kg km^−2^). Halliday et al. (2014) found a similar allocation in a WWTP influenced rural catchment, with 78% of total P being present as dissolved P. Assuming the WWTP is the main source for oPO_4_ − P and NH_4_ − N, it might account for at least 48% and 65% of total N and P export respectively. This is higher compared to many assumptions of other studies (Ehrhardt et al., 2019; Alexander et al., 2007; Basu et al., 2010) made for bigger catchments. That also implies that, in comparison to agricultural derived phosphorus, which can be more easily held back by sedimentation, WWTPs introduce a high share of mobile and bioavailable phosphorus (Millier und Hooda, 2011; Neal et al., 2010), which might be a bigger threat further downstream.

One question arising is whether a more stringent nutrient treatment could solve that problem. However, our data suggest that excessive water volumes lead to decreasing cleaning efficiencies. As long as this is the case, a high share of nutrients will probably still be exported during events. Adjusting the stormwater management could be more beneficial. By extending for example sewer storage capacities or installing separate sewer systems, a reduced flow through the WWTP during and after events, as well as a reduction of the number of CSO spills, could be achieved.

### CSO events

The modified conductivity loggers indicated a total of 27 discharge events from the sewer overflows, so CSO events accounted for 35% of all events. Precipitation amounts ranged from 1.2 to 55 mm (mean 11.5 mm), with mean intensities of 2.2 mm h^−1^. In comparison to other studies, less intensity and slightly less total precipitation amounts seem to cause a CSO event: Phillips et al. (2012), who monitored a middle-sized WWTP (30,000 PE), counted 36 events for a period of 12 months, with rainfall ranging from 2 to 41 mm. In comparison, Sandoval et al. (2013) monitored 22 events in Berlin, with average rainfall intensities of 4.6 mm h^−1^ and mean rainfall amounts of 12 mm. Due to the small size of the village and no separate storage for excessive water besides one storage canal, the storage capacity of the SS is reached very fast, leading to CSO events with comparatively low rainfall intensities and lower rainfall amounts.

Most of the CSO events occurred during early spring (JFM) when soils were saturated and during summer (JAS), when precipitation amounts and intensities were the highest. During these periods, when the SS is still close to its maximum storage capacity from previous events, small rainfall amounts can lead to a CSO event, which was also found in other catchments (Sandoval et al., 2013; McGrath et al., 2019; Lawler et al., 2006; Gallé et al., 2018).

**Fig. 6:**
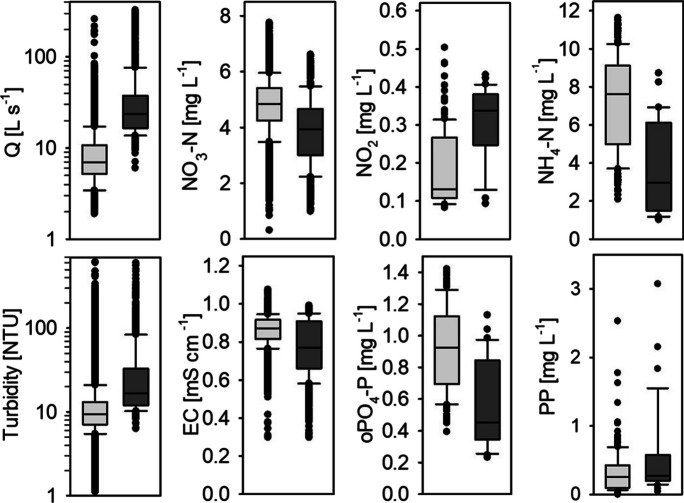
Water quantity and quality during ‘normal’ events (light grey) and during periods, when CSO discharge is active (5 monitored events) (dark grey); *CSO*, combined sewer overflow; *PP*, particulate phosphorus

**Fig. 7:**
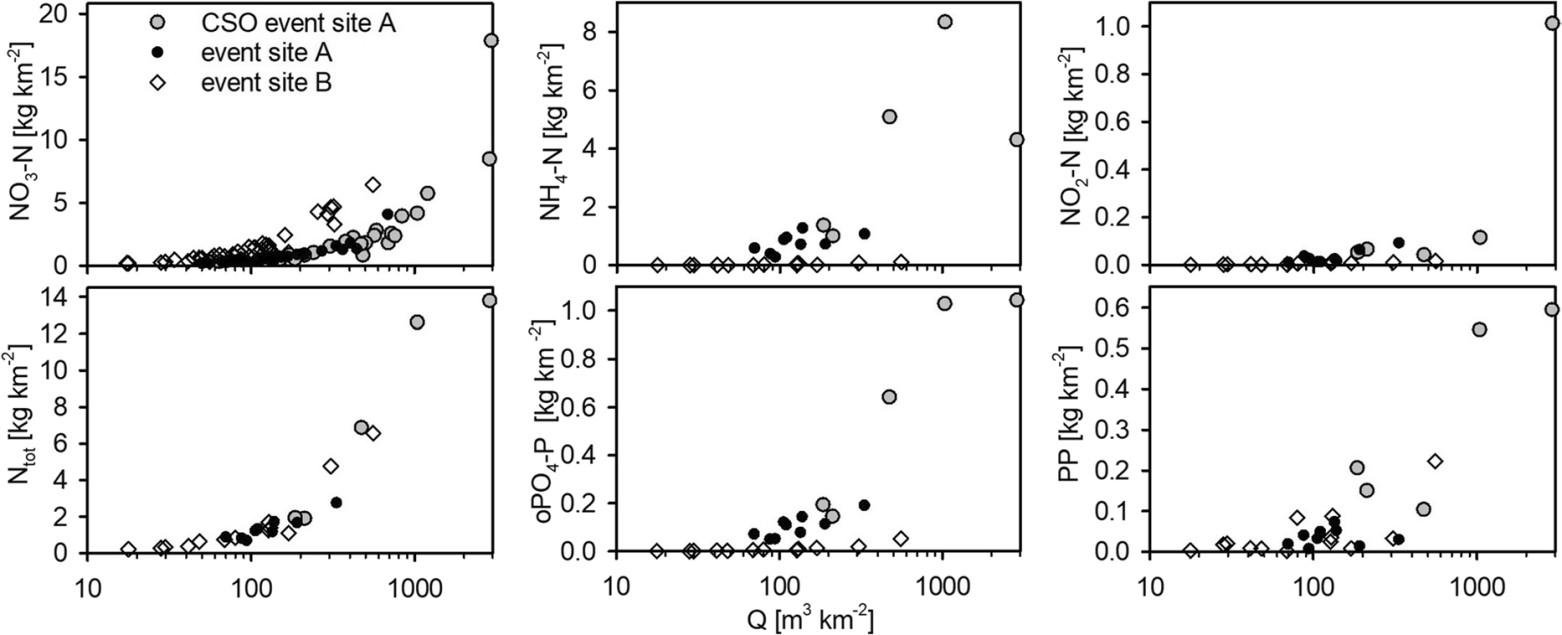
Total event discharge in relation to total nutrient export for both sites and for CSO events; *CSO*, combined sewer overflow; *PP*, particulate phosphorus

NH_4_ − N concentrations during CSO events on site A were in similar range than NH_4_ − N measurements in higher urbanised areas, which reached from 6.25 mg L^−1^ (Lawler et al., 2006) up to 10.5 mg L^−1^ (Gallé et al., 2018). Median discharge increased significantly (Fig. 6) from 6 to 25 L s^−1^. oPO_4_ − P, NH_4_ − N, and NO_3_ − N concentrations were diluted more strongly compared to events, where no CSO spilling occurred. However, due to the increased discharge, total exported loads where higher during CSO events (Fig. 7). Phillips et al. (2012) made similar observations: discharge which exceeded the design capacity of the WWTP and thus was led to the CSO contributed a disproportional share of nutrient load export, ranging from 20 to 45%. Median NO_2_ − N concentrations, however, increased, while loads were not clearly higher, except for one event.

EC decreased more pronounced during CSO discharges, supporting the assumption that during “normal” events, a high share of wastewater reaches the stream. PP and turbidity, however, show a different behaviour. During CSO events, the concentrations and loads were the highest. Highly polluted sediments can be built up within SSs during dry periods and mobilised as soon as the water table rises (Lawler et al., 2006). Climate change might support this effect, as heavy rainfalls as well as longer dry periods are expected to rise (Trenberth, 2011), leading to more sediment build up and thus even higher loads exported during events. At the same time, baseflow stream discharges might get even lower during long-lasting dry periods. This combination might lead to higher in-stream concentrations due decreased dilution effects (Sandoval et al., 2013; Phillips et al., 2012).

Enhanced peak discharges may also decrease the instream retention of nutrients. Especially catchments with fast travel times were found to react very sensitive even to small inputs of wastewater (Gardner et al., 2011), as there is less time for (de-)nitrification, biological assimilation, or sorption processes. CSO events and the discharge from small WWTPs decrease the catchment response time significantly. This leads to a fast transport to lower river reaches and increases the risk for an accumulation of nutrients in the receiving rivers.

## Conclusions

With our unique measurement setup in two neighbouring catchments, we were able to show that even small-sized sanitary infrastructure increases the complexity of a catchment. Storage effects and nutrient processing within these structures alter water quantity and quality significantly, leading to a high input of phosphorus and nitrogen. There are several key points which can be drawn from the presented data, which should be taken into account, when sanitary infrastructure is present in catchments.

(i). Introduction of reactive nutrient factions:

- In agricultural catchments, NO_3_ − N and PP are the dominating nitrogen and phosphorus fractions. Point sources, however, may introduce oPO_4_ − P, NH_4_ − N, and NO_2_ − N, which can be indirect source for PP and NO_3_ − N.

(ii). Events are highly relevant for nutrient export

- Sealed areas and sewer systems decrease the response time of a catchment, leading to a higher volumetric water throughput. At the same time, fraction of reactive nutrient fractions tend to increase, presumably due to the release of poorly treated wastewater, leading to high nutrient loads during events.

(iii). Variable influence of WWTP

- Water quality of point sources may change throughout the year and during events. Assuming constant water quantity and quality from point sources might, therefore, be a too simplistic approach in load estimations or modelling studies, which might underestimate the export of nutrients, especially when water quality is not monitored on a high-frequency basis.

(iv). Influence of CSO

- CSO events lead to a dilution of dissolved nutrients, while PP concentrations and turbidity are elevated during this period.
- Despite the dilution, nutrient loads are still higher during events, making CSO events to a “hot moment” for nutrient export.

Despite the small size of the settlement, the limited storage capacity of such SSs and small WWTPs might play a key factor in nutrient export, which should not be neglected when analysing rural areas. To better understand storage effects, nutrient transformation processes, and the influences of different nutrient sources, further analyses have to be performed, which are based on single events and take more data from the WWTP into account.

### Supplementary Information

ESM 1 (DOCX 317 kb)

## Data Availability

The data sets generated and/or analysed during the present study are available from the corresponding author on reasonable request.
