# Supplementary material for: Small villages and their sanitary infrastructure—an unnoticed influence on water quantity and a threat to water quality in headwater catchments
Source: Environ Monit Assess. 2023 Nov 16;195(12):1482. doi: 10.1007/s10661-023-12051-6 (PMC10654200; doi:10.1007/s10661-023-12051-6)
Supplement: Supplementary file 1 — (DOCX 317 kb) [file 10661_2023_12051_MOESM1_ESM.docx]

**Supplementary Information**

**Small Villages and their Sanitary Infrastructure – an unnoticed Influence on Water Quantity and a Threat to Water Quality in Headwater Catchments**

Caroline Spill^1*^, Lukas Ditzel^1^, Matthias Gassmann^1^

*^1^**Department Hydrology and Substance Balance, University of Kassel, Germany*

^*^Corresponding author: caroline.spill@uni-kassel.de

Table 1S Uncertainty ranges of laboratory nutrient measurements

| Nutrient | method | device | type | manufacture | Precision [% from measured value] |
| --- | --- | --- | --- | --- | --- |
| $\mathbf{NO}_{\mathbf{3}}\mathbf{-N}$ [$\mathbf{mg}\mathbf{L}^{\mathbf{-1}}$] | DIN EN ISO 10304-1 | IC | Compact IC 761 | Metrohm | 10 |
| $\mathbf{NH}_{\mathbf{4}}\mathbf{-N}$ [$\mathbf{mg}\mathbf{L}^{\mathbf{-1}}$] | DIN 38406-5 | UV-VIS | Cary 100 | Agilent | 5 |
| $\mathbf{NO}_{\mathbf{2}}\mathbf{-N}$ [$\mathbf{mg}\mathbf{L}^{\mathbf{-1}}$] | DIN EN 26777 | UV-VIS | Cary 100 | Agilent | 10 |
| $\mathbf{o}\mathbf{PO}_{\mathbf{4}}\mathbf{-P}$ [$\mathbf{mg}\mathbf{L}^{\mathbf{-1}}$] | DIN EN ISO 6878 | UV-VIS | Cary 100 | Agilent | 5 |
| $\mathbf{P}_{\mathbf{tot}}$ [$\mathbf{mg}\mathbf{L}^{\mathbf{-1}}$] | DIN EN ISO 6878 | UV-VIS | Cary 100 | Agilent | 7 |

**Table 2S** date of sampled events, number of samples and Q_max_ of the sampled event for site A and site B; grey highlighted events are events which were sampled on the same date.

|  | Kelze (site A) | | |  | Nesselbach (site B) | |
| --- | --- | --- | --- | --- | --- | --- |
| date | No. Samples | | Q_max_ (l/s) |  | No. Samples | Q_max_ (l/s) |
| 28.01.2021 | 25 | | 65 |  | - | - |
| 11.04.2021 | - | | - |  | 24 | 25 |
| 10.05.2021 | 24 | | 43 |  | 24 | 43 |
| 18.05.2021 | 5 | | 30 |  | 7 | 11 |
| 19.05.2021 | - | | - |  | 13 | 16 |
| 25.05.2021 | - | | - |  | 6 | 18 |
| 10.06.2021 | 24 | | 61 |  | 10 | 18 |
| 22.06.2021* | 3 | | 13 |  | - | - |
| 24.06.2021 | - | | - |  | 14 | 13 |
| 29.06.2021 | - | | - |  | 6 | 17 |
| 01.07.2021 | - | | - |  | 19 | 12 |
| 06.07.2021 | - | | - |  | 10 | 14 |
| 25.07.2021 | 8 | | 20 |  | - | - |
| 26.07.2021 | 5 | | 28 |  | - | - |
| 31.07.2021* | 3 | | 13 |  | - | - |
| 01.08.2021 | 8 | | 41 |  | - | - |
| 22.08.2021 | - | | - |  | 7 | 7 |
| 25.08.2021* | 12 | | 12 |  | - | - |
| 11.09.2021 | 15 | | 58 |  | 5 | 18 |
| 21.10.2021 | 11 | | 26 |  | - | - |
| 03.11.2021 | 24 | | 16 |  | - | - |
| 04.11.2021 | 7 | | 7 |  | - | - |
| 08.11.2021 | 18 | | 15 |  | 5 | 12 |
| 30.11.2021 | 13 | | 12 |  | - | - |
| 01.12.2021 | 6 | | 12 |  | 8 | 12 |
| 02.01.2021 | 14 | | 60 |  | 10 | 23 |
| 04.01.2022 | 24 | | 20 |  | - | - |
| 28.01.2022 | - | | - |  | 4 | 9 |
| 01.02.2022 | - | | - |  | 20 | 13 |
| 06.02.2022 | 12 | | 50 |  | 19 | 20 |
| sum of events (without *) | 17 | |  |  | 18 |  |
| Events with same sample date |  | 8 | | | | |
| Mean Q_max_ |  | | 30.1 |  |  | 16.5 |
| Mean Q_max_ of all events |  | | 33.6 |  |  | 18 |
| *no event but increase in discharge and NO3-N concentration | | | | | |  |


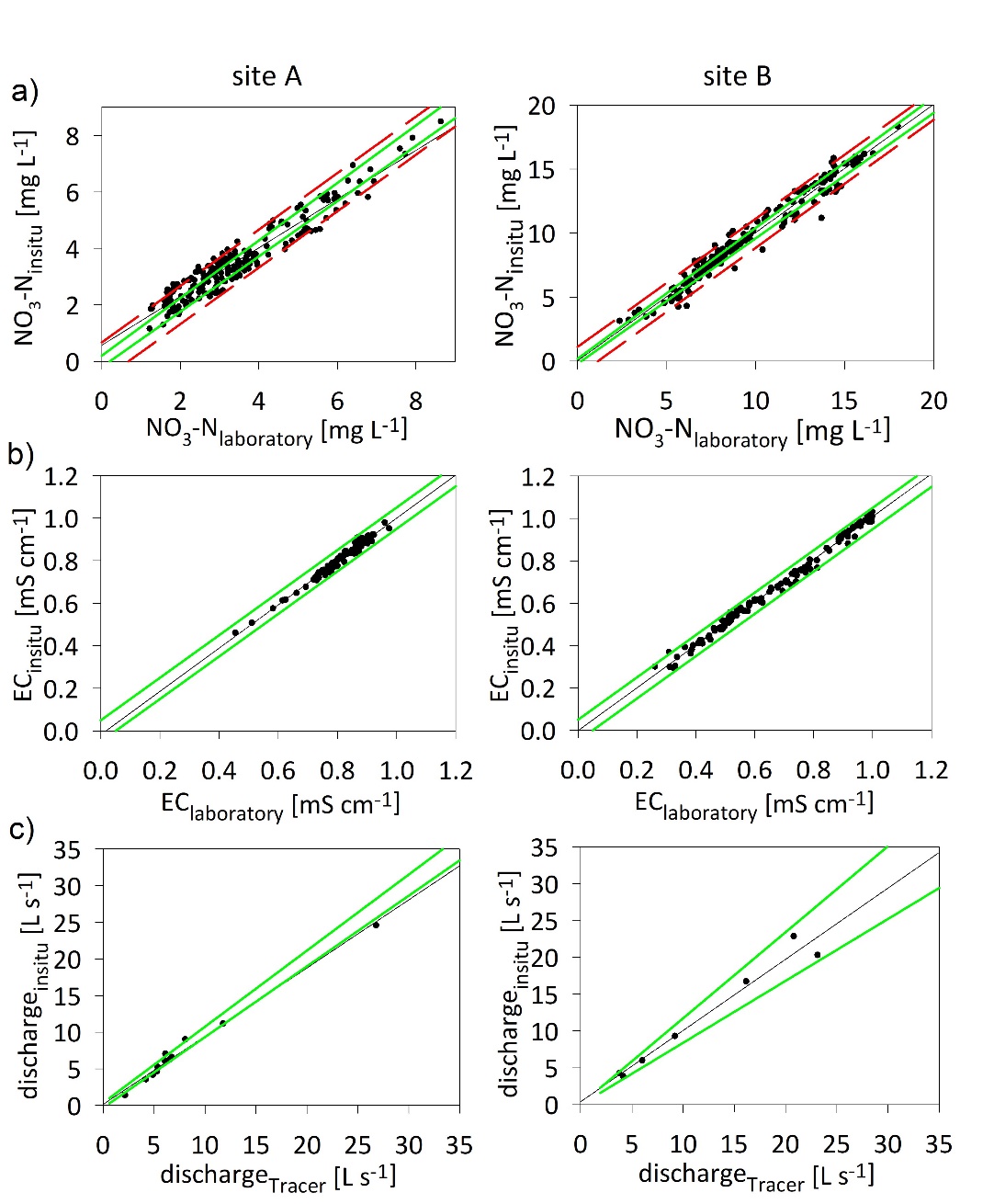
**Fig. 3S** Relationship of (a) in-situ measurements and laboratory analysis of $\mathrm{NO}_{3}$, (b) electrical conductivity (EC) and (c) discharge calculated from water levels (site A) and by the magnetic inductive discharge probe (site B) and discharge calculated by salt tracer experiments. Black lines represent the linear regression, green lines indicate the uncertainty specified by the manufacturers and the red lines represent estimated uncertainty bounds, when manufacturers specification were found to be too optimistic.
